# Aggression and its predictors among elementary students

**DOI:** 10.5249/jivr.v11i2.1102

**Published:** 2019-07

**Authors:** Nooshin Salimi, Akram Karimi-Shahanjarini, Forouzan Rezapur-Shahkolai, Behrooz Hamzeh, Ghodratollah Roshanaei, Mohammad Babamiri

**Affiliations:** ^*a*^Department of Public Health, School of Public Health, Hamadan University of Medical Sciences, Hamadan, Iran.; ^*b*^Social Determinants of Health Research Center, Hamadan University of Medical Sciences, Hamadan, Iran.; ^*c*^Research Center for Health Sciences, Hamadan University of Medical Sciences, Hamadan, Iran.; ^*d*^Research Center for Environmental Determinants of Health, Kermanshah University of Medical Sciences, Kermanshah, Iran.; ^*e*^Modeling of Noncommunicable Diseases Research Center, Hamadan University of Medical Sciences, Hamadan, Iran.; ^*f*^Department of Biostatistics, School of Public Health, Hamadan University of Medical Sciences, Hamadan, Iran.; ^*g*^Department of Ergonomics Center, School of Public Health, Hamadan University of Medical Sciences, Hamadan, Iran.

**Keywords:** Violence, Aggression, Victimization, Primary school

## Abstract

**Background:**

Aggression is the most significant psychopathological risk factor. It is a multifaceted construct and can affect students’ social, mental and physical health. The present study was conducted to investigate factors associated with aggression among elementary school girls and boys using the theoretical framework of Social Cognitive Theory in Kermanshah city, Iran.

**Methods:**

The cross-sectional study was conducted on 900 students, including 445 girls and 455 boys, 563 parents and 104 elementary school teachers in the fourth, fifth and sixth educational grades in Kermanshah city in 2018. The proportionate stratified random sampling was used. The Adolescent Peer Relations Instrument (APRI) was used to measure aggression. To measure the variables of social cognitive theory, a researcher-made questionnaire was used. Data analysis was conducted using descriptive and inferential statistics via the SPSS software.

**Results:**

It was showed that 29% and 10% of the students had moderate and high levels of aggression, respectively. Also, 30.6% of them were moderate victims and 45.6% were intense victims. Self-efficacy (p less than 0.001), perceived social norms (p=0.011), observational learning (p less than 0.001), outcome expectations (p=0.027), outcome expectancies (p=0.028) and per-ceived situational (p less than 0.001) were reported as the significant predictors of aggressive behaviors in the students based on the Social Cognitive Theory constructs. In total, they explained for 37.3% of changes in aggressive behaviors. Parents’ knowledge (p=0.005), parents’ attitude (p=0.012), teachers’ attitude (p less than 0.001), and teachers’ self-efficacy (p=0.021) had statistically significant relationships with aggression in the students.

**Conclusions:**

Aggression among children and adolescents is getting alarmingly prevalent. The Social Cogni-tive Theory is the appropriate framework for the prediction of aggression behaviors in children and adolescents. Therefore, designing and implementing educational interventions based on this theory can help with the management of aggression in children and adolescents.

## Introduction

Aggression is the most significant psychopathological risk factor among children and adolescents. Aggression has a multifaceted construct and can affect the social, psychological and physical health of students and teachers. In social psychology, aggression refers to behaviors emerged to harm another person. ^1,2^ The definition of aggression falls into two categories as instrumental aggression and hostile aggression. While instrumental aggression is used to achieve an external goal, hostile aggression intends to harm others and includes two forms of explicit aggression (physical and verbal aggression) and social aggression.^3^ Physical aggression refers to purposefully harming someone to cause pain such as beating, pushing, throwing objects, etc.^4,5^ Application of the aggressive language such as yelling and screaming that causes hurting emotions and credit of a person and lowers a person’s value is called verbal aggression. ^5,6^ Social aggression refers to actions to harm others’ social statuses and friendships.^7^ Heavy silence and negligence are some examples of social aggression.^5,8^ Aggression is prevalent among children and adolescents.^9,10^ Approximately, one in every 10 children suffer from chronic aggressive behaviors or is harassed by peers.^11^ In 2018, the UNESCO estimated that approximately 30% of all students annually experienced some type of aggression at school.^12^

No appropriate data is available on the degree of aggression among Iranian schoolers. However, there are some statistics in the different parts of Iran. For example, according to a study in Tehran city, 14.2% of elementary school students were aggressive.^13^ A study in Kermanshah showed that the prevalence of aggressive behaviors in middle school was from 13.7 to 52.1 percent in boys and from 10.2 to 64.6 percent in girls.^14^ Studies in different countries have shown an increase in the prevalence of aggressive behaviors in children aged 11 to 13 years.^15,16^ Hence, prevention and reduction of aggression in students before this age are recommended. ^17^

Due to physical, psychological and social complications of aggression, attention of researchers to this important subject has been increased. Aggression causes devastating effects on social competence, efficiency, and interpersonal relationships ^18-20^ and can develop a negative image among peers and teachers, peer rejection, academic underachievement, alcohol abuse, drug abuse, delinquency, suicide, self-harm, aggression, and anxiety.^21-23^ It is believed that aggressive behaviors in childhood is associated with an increased risk of psychological problems in adolescence and adulthood.^24^ In addition to the individual dimensions of aggression and its consequences in the school, it can have extensive social and economic costs. Research indicates that school violence is associated with less employment and a further use of mental health services in adulthood.^25,26^

The Social Cognitive Theory provides explanations on how aggressive behaviors are developed through the description of how people learn aggression through observational learning and reinforcement. Recently, researchers have focused on the Social Cognitive Theory to explain aggression in children.^27-29^ According to the social cognitive theory, children and adolescents who are exposed to aggressive and violent behaviors experience a higher rate of violence than other children and adolescents.^6^ Also, friends, parents and teachers act as role models, and child behaviors is shaped through the modeling of their positive or negative communication skills.^30,31^

Supporting individuals with their aggression behaviors and their beliefs on the positive and negative consequences of such behaviors can shape violence.^32,33^ The use of Social Cognitive Theory as a theoretical framework is useful for investigating factors affecting aggression in students. The present study aimed to investigate factors associated with aggression among elementary school girls and boys in Kermanshah city, Iran using the theoretical framework of the social cognitive theory.

## Methods 

This cross-sectional study was conducted on 900 students including 445 girls and 455 boys, 563 parents and 104 elementary school teachers in the fourth, fifth and sixth educational grades in Kermanshah city in 2018. 

Kermanshah encompasses 1.5% of the whole country and with a population of about 2 million people in 2016 is located in the west of Iran, and is bordered by Iraq. In terms of human development indicators including income, education, and health, Kermanshah ranks the13th out of 28 provinces in 2009.^34^ Regarding the health status, Kermanshah is considered one of the deprived provinces in Iran.^35^

In this study, the proportionate stratified random sampling method was used. The stratifications were selected based on the students’ gender, school’s status in terms of implementing the program of health promoting schools as health promoting and non-health promoting schools and the urban area as favored, less-favored and non-favored. The list of schools and number of the fourth to sixth grade elementary students in Kermanshah city were collected from the General Education Office. Using the simple random sampling method, the required sample was selected in terms of number of students and schools in each class, with the consideration of different socioeconomic statuses. Inclusion criteria to recruit the samples were studying in elementary schools in the fourth, fifth and sixth educational grades, studying at the schools of Kermanshah city, lack of any acute mental health problems based on the health profile, and parents' consent for the participation of their children.

A researcher-made questionnaire also was used to measure the constructs of the social cognitive theory, which was developed based on previous studies.^36-40^

The Adolescent Peer Relations Instrument (APRI) was used to measure students’ aggression. It consists of 36 items with the aim of measuring the specific aggression behavior and developed victimization in the peer group of adolescents in different dimensions including verbal aggression, social aggression, physical aggression, verbal victimization, social victimization and physical victimization. It has a 6-point Likert scale from 1 (never) to 6 (everyday). The Cronbach’s alpha coefficient for the whole questionnaire was reported as 0.89.41 Validity and reliability of this questionnaire were also confirmed in Iran^42^ While this questionnaire lacked a cut-off point, using the profile class method and Mplus software the cut-off points was determined in three dimensions as aggressor, victim and both (aggressor and victimized) and at three levels of low, medium and high in this study.

We developed a questionnaire to measure the determinants of students’ aggregation. It includes knowledge about the type of aggressive behaviors and their consequences consisting of 11 questions (is the negligence a part of aggression?), self-efficacy to control own aggression with 7 questions (I can control myself when someone harasses me, and I do not beat him), social support to control aggression with 7 questions (when I get angry, my friends calm me down), perceived social norms about aggressive behaviors with 12 questions (my parents believe that I should fight with others wherever necessary), observational learning of aggressive behaviors through observing the aggressive behaviors of friends, parents, teachers and the media with 10 questions (when I see a violent film or cartoon, I become violent like a film or cartoon character), outcome expectations about the consequences of aggressive behaviors and outcome expectancies about the value which the student place on related consequences, each with 5 questions (if I am aggressive, others will accept me/it is important for me that others accept me) and perceived situation about aggression with 7 questions (in our school, fighting between students is common). To measure factors in parents and teachers, it included knowledge about types of aggressive behaviors and their consequences with 11 questions, and attitude toward children or students’ aggressive behaviors with 8 questions (it is natural that students mock each other) and self-efficacy to reduce their children or students' aggressive behaviors in difficult conditions with 5 questions (I can prevent my children or students from aggression in any situation), with a 5-point Likert scale.

The Content Validity Index (CVI) and Content Validity Ration (CVR) were used to evaluate the data collection tool in terms of content and face validity. Opinions of 15 specialists in health education and promotion and 1 specialized psychologist were sought. The content validity ratio for all constructs was higher than 0.8 and for content validity was more than 0.9. The internal consistency method was also used to measure its reliability. The pilot questionnaire was filled out by 90 elementary school female and male students in Kermanshah city, 54 parents and 15 teachers. They were not included in the original sample recruitment. The Cronbach alpha coefficient of the students' questionnaire for self-efficacy was reported as 0.77, social support was 0.72, perceived social norms was 0.81, observational learning was 0.87, outcome expectations was 0.70, outcome expectancies was 0.72, and perceived situational was 0.73%. Also, the Cronbach's alpha coefficient in the parents' questionnaire for the attitude was reported as 76%, self-efficacy was 0.82. In the teachers' questionnaire for the attitude was reported as 0.76 and self-efficacy was 79%. 

This study was conducted after coordination with the Education Office authorities in the province and schools. Also, the informed consent form was signed by the parents.

The students, parents, and teachers filled out the final questionnaire within 1 month. The necessary explanations were provided by the researcher on how to answer the questions. Data was analyzed using descriptive and inferential statistics via the SPSS software v.16 and independent t-test, analysis of variance, correlation analysis, and linear regression. The significance level was set as p<0.05.

## Results

All parents signed the informed consent form. The mean age and standard deviation (SD) of the students were 11.16±0.96 years. The majority of them were male (50.6%). Of the students, 32.9% and 67.1% were studying in health promoting schools and non-health promoting schools, respectively. The parents mostly had a diploma education degree and the average number of children in the family was 2.31±1.01 people. The students (79%) used the Android and video games for 1-7 hours with a mean of 1.48 hours per day. Also, 13.6% of their mothers were employee and 86.4% were housewives (Table 1). 61% of the students had a low level of aggression. Besides, 29% and 10% of them had moderate and high levels of aggression. For victimization, 23.8% were at a low level, 30.6% at the moderate level and 45.6% at the high level. Both aggressiveness and victimization at a high level was available in 20.3% of the samples. Using the independent t-test results, the rate of physical, verbal and social aggression in the male students was significantly higher than the female students (p<0.001), but the rates of verbal (p = 0.082) and social victimization (p=0.164) had insignificant differences between the female and male students, and only the rate of physical victimization (p= 0.20) was higher in the males than females (Table 2). Of demographic factors, age (p<0.001), the education level (p<0.001), gender (p<0.001), duration of the use of Android and video games (p<0.001), residence area in terms of the socioeconomic status (p<0.001), rate of family income (p=0.002), father's education level (p= 0.022), and mother's education level (p=0.027) were significantly related to aggression. Accordingly, the increase of age, higher education level, male gender, duration of the use of Android and video games, living in an area with low socioeconomic welfare, low income of families and low education of parents had statistically significant relationships with the increase of aggression in the students. Meanwhile, the mother's employment status (p=0.067), number of children (p=0.126), type of school in terms of being health promoting and non-health promoting (p=0.066) and type of life with the parent such as living with parents, father or mother (p=0.280) had no statistically significant relationships with aggression in the students. Of the demographic variables, age (p=0.008), place of residence in terms of the socioeconomic status (p<0.001) and the type of school in terms of being health promoting and non-health promoting (p<0.001), had statistically significant relationships with the rate of victimization. Accordingly, the rate of victimization was associated with older students, lower socioeconomic welfare and non-health promoting schools. Also, gender (p=0.490), the mother's employment status (p=0.443), father's education level (p=0.867), mother's education level (p=0.191), duration of the use of Android and video games (p=0.941) and number of children (p=0.457) did not have statistically significant relationships with victimization.

**Table 1:** The demographic factors and their relationship with the mean of students' aggression.

Variables		Number	Percentage	Mean± (SD) of students' aggression	P- value
**Gender**	female	445	49.4	28.89±(11.424)	p<0.001
	male	455	50.6	35.66±(16.561)	
**Age**	9 Years	22	2.4	25.27±(6.096)	p<0.001
	10 Years	211	23.5	27.55±(10.734)	
	11 Years	345	38.4	32.34±(14.717)	
	12 Years	244	27.1	35.96±(15.908)	
	13 Years	77	8.6	35.88±(16.826)	
**Type of school**	Health Promoting Schools	296	32.9	31.47±(12.178)	p=0.066
	Non-Health Promoting Schools	604	67.1	32.73±(15.704)	
**Father's education level**	Illiterate	16	2.9	34.94±(18.502)	p=0.022
	Elementary	68	12.2	35.82±(15.890)	
	Middle School	112	20.1	35.02±(16.987)	
	Diploma	187	33.6	31.86±(13.918)	
	Collegiate	174	31.2	30.18±(13.719)	
**Mother's education level**	Illiterate	25	4.4	39.68±(20.477)	p=0.027
	Elementary	101	17.9	34.47±(16.848)	
	Middle School	109	19.4	33.08±(14.300)	
	Diploma	207	36.8	32.04±(14.518)	
	Collegiate	121	21.5	30.02±(13.583)	
**Daily duration of the android and video games**	<1 hour	523	61.2	30.21±(13.701)	p<0.001
	1-2 hour	197	23.0	33.31±(13.988)	
	2-3 hour	65	7.6	34.60±(12.318)	
	3-4 hour	30	3.5	34.33±(13.042)	
	>4 hour	40	4.7	44.88±(20.048)	
**Residence area in terms of socioeconomic status**	Favored	289	32.1	29.51±(12.275)	p<0.001
	Less-favored	299	33.2	32.47±(14.269)	
	non-favored	312	34.7	34.76±(16.490)	
**Mother's employment**	Employed	106	13.6	31.18±(12.782)	p=0.067
	Unemployed	674	86.4	32.90±(15.091)	
**Type of life with the parent**	With parents	745	94.5	32.50±(14.629)	p=0.280
	Mother or father	43	5.5	34.40±(15.792)	
**Number of children in the family**	1	134	16.6	31.16±(14.421)	p=0.126
	2	412	51.2	32.36±(14.161)	
	3	181	22.5	34.33±(15.053)	
	≥4	78	9.8	34.40±(17.743)	

**Table 2:** Mean, Standard Deviation and comparison of physical, verbal and social aggression and victimization among the girls and boys.

Types of aggression	Sex	N	Mean	Std. Deviation	t-test sig
**Physical aggression**	female	445	9.09	4.385	p<0.001
	male	455	13.42	7.066	
**Verbal aggression**	female	445	9.51	4.062	p<0.001
	male	455	11.41	5.614	
**Social aggression**	female	445	10.29	4.411	p<0.001
	male	455	10.83	5.531	
**Physical victimization**	female	445	11.58	6.102	p=.020
	male	455	13.37	6.611	
**Verbal victimization**	female	445	13.69	6.728	p=.082
	male	455	15.07	7.199	
**Social victimization**	female	445	12.10	6.731	p=.164
	male	454	12.02	6.119	
**Total aggression**	female	445	28.89	11.424	p<0.001
	male	454	35.66	16.561	
**Total victimization**	female	445	37.37	18.092	p=.490
	male	454	40.38	17.964	

According to the correlation analysis, except for knowledge (r=-0.039, p=0.241), there were statistically significant relationships between other constructs of the social-cognitive theory and aggression in the students (Table 3). To assess the aggression predictors in the students, the linear regression analysis was conducted (Table 4). Except for the knowledge and social support to control aggression construct, other constructs of the social-cognitive theory predicted 37.3% of changes in aggression. Parent's knowledge (p=0.005), parent's attitude toward their children aggressive behaviors (p=0.012), teacher's attitude toward their students' aggressive behavior construct (p<0.001), and teacher's self-efficacy to reduce their students' aggressive behaviors construct (p=0.021) were significantly associated with aggression. Therefore, parents with lower knowledge and higher attitude scores had more aggressive children, and teachers with higher attitude and lower self-efficacy scores had more aggressive students. The self-efficacy to reduce their child aggressive behaviors in parents construct (p=0.397) and knowledge in teachers construct (p=0.267) were not significantly associated with aggression in the students.

**Table 3:** The correlation between constructs of Social Cognitive Theory and aggression in students.

	Aggression	Knowledge	Self-efficacy	Social support	Perceived social norms	Observational learning	Perceived situational	Outcome expectancies	Outcome expectations
**Aggression**	1	-.039	-.386**	-.143**	.386**	.532**	-.235**	.301**	.385**
**Knowledge**		1	.122**	.166**	-.028	-.036	.051	-.048	-.001
**Self-efficacy**			1	.187**	-.162**	-.293**	.157**	-.196**	-.250**
**Social support**				1	-.155**	-.178**	.163**	-.067*	-.138**
**Perceived social norms**					1	.570**	-.204**	.294**	.455**
**Observational learning**						1	-.181**	.366**	.547**
**Perceived situational**							1	-.120**	-.081*
**Outcome expectancies**								1	.400**
**Outcome expectations**									1

*. Correlation is significant at the 0.05 level (2-tailed). **. Correlation is significant at the 0.01 level (2-tailed).

**Table 4:** Regression Analysis to predict aggressive behavior based on the structures of Social Cognitive Theory.

Target group	Determinants	Mean	Std. Deviation	Standardized Coefficients Beta	t	Sig.	R Square
**Students**	(Constant)				7.962	.000	**0.373**
	Knowledge	6/33	2/225	.005	.192	.848	
	Self-efficacy	24/69	7/053	-.230	-8.003	.000	
	Social support	23/97	4/856	.006	.230	.818	
	Perceived social norms	25/28	9/149	.086	2.564	.011	
	Observational learning	21/28	9/074	.332	9.149	.000	
	Perceived situational	18/80	4/671	-.111	-3.993	.000	
	Outcome expectancies	18/46	4/542	.075	2.207	.028	
	Outcome expectations	12/51	4/371	.066	2.214	.027	
**Parents**	Knowledge	7/49	1/910	-.119	-2.811	.005	**0.032**
	Self-efficacy	20/17	3/317	-.036	-.849	.397	
	Attitude	32/29	5/411	.106	2.514	.012	
**Teachers**	Knowledge	7/17	2/017	-.037	-1.111	.267	**0.068**
	Self-efficacy	19/60	3/167	-.089	-2.307	.021	
	Attitude	30/53	4/857	.293	7.770	.000	

## Discussion

This study indicated that of the students, 29% had a moderate level of aggression and 10% had a high level of aggression. Regarding being victim, 30.6% of the students were at a moderate level and 45.6% were at a high level. Various studies have shown that children and adolescents are affected by school violence with a range from less than 10% to over 65%.^16,43^ In addition to cultural differences in any society, differences in results can be related to various tools and methods for the measurement of aggression. In the present study, physical, verbal and social aggression in males was significantly higher than that of females. In regard to victimization, the results showed that, except for physical victimization, there were no significant differences between male and female students. The results of various studies indicated that males were more exposed to physical and verbal violence than females. While in the females, social aggression was more common than males.^16,44^ According to the results of this study, an increase in age was significantly associated with the increase of aggression and victimization in the students. Increase of age has a significant relationship with aggression under the age of 15 years, which is consistent with the results of our study.^44,45^

In the present study, duration of the use of Android and video games was directly and significantly associated with aggression. Accordingly, the more hours of the use of electronic devices might be associated with negligent or permissive parenting, and consequently aggression. Additionally, some video games might be violent video games, and could be associated with aggressive behaviors.^46^ A large body of research in the world has indicated that an increase in the duration of the use of Android and video games would increase the rate of aggression.^47-49^ The results of our study indicated that the area of residence in terms of the socioeconomic status has a significant relationship with aggression in the students, i.e., the prevalence of aggression in non-favored and less-favored areas would be higher than favored areas. There were no statistically significant differences between aggression in non-favored and less-favored areas, which could be due to economic problems and poverty in families, as well as high levels of violence in areas with a low socioeconomic status. Research indicates that socially and economically deprived children and adolescents often face more stress, discrimination, and insults in the school. Poverty can aggravate the lack of self-confidence that lays the groundwork for further bullying and victimization in such areas.^16^ The parents' level of education had a significant relationship with aggression in the students. Although a few studies investigated the relationship between parent's education and aggression in children, some indicated that high levels of parent's education were associated with lower levels of aggressive behaviors in children.^50,51^

In this study, constructs of the Social Cognitive Theory predicted 37.3% of changes in aggression. This result is somewhat similar to those of studies conducted using other social cognitive models such as the theory of planned behavior. For example, Alimoradi et al. reported that constructs of the theory of planned behavior predicted 36.3% of verbal aggression and 21.1% of physical aggression.^52^ Another study in Iran indicated that the theory of planned behavior predicted 20% of changes in aggressive behaviors.^53^ Also, the Heirman et al. ‘s study showed that the theory of planned behavior could predict 30% of cyber aggression. ^54^ According to the theory of planned behavior, the intention was the main determinant of behavior influencing attitude, subjective norms, and perceived behavioral control.^55^

In this study, the students' and teachers' knowledge on aggressive behaviors had no significant relationships, but a significant relationship was found between the parents' knowledge of aggression in students. Conversely, some studies showed a significant relationship between students' knowledge and the rate of aggression.^56,57^ Given the comparison of teachers’ knowledge scores of parents and students and their level of education toward two groups, teachers lacked a high level of knowledge toward the types of aggression. Similar to our study, various studies suggested that most teachers lacked a clear understanding of the definition of expression.^58,59^ Other studies indicated that increasing the knowledge of parents had a significant relationship with the reduction of aggression in children, ^60,61^ which was consistent with our study.

Aggressive adolescents may expect different outcomes of their aggression such as gaining more popularity among peers.^27,62^ In this study, outcome expectations of the consequences of aggressive behaviors and outcome expectancies about the value which the students placed on these consequences had a significant relationship with aggression, i.e. the more students considered the aggression outcomes positive and significant, the higher was their aggression. Zavareh et al. indicated a significant relationship between outcome expectations and aggression. Other studies indicated a positive and significant relationship between outcome expectancies and aggression, that was consistent with the results of our study.^56,63^

There is a significant relationship between the parents and teachers’ attitude toward their children or students’ aggressive behaviors, and aggression in our study. In this regard, Erdoğdu et al. found a significant relationship between the attitude of parents and teachers, and aggression in the students.^64^

In this study, observational learning of aggressive behaviors was the strongest construct for predicting aggression in the students. Various studies indicated that observational learning had the most effect on aggressive behaviors.^27^ The Iranian study by Omidi et al. reported a direct and significant relationship between aggressive behaviors of adolescents and aggressive behaviors in the family, watching the police and aggressive movies.^65^

According to the Social Cognitive Theory, this study indicated a significant relationship between perceived situational and aggression in the students, in other words, aggressive behavior occurred less frequently among students who perceived their school as a safe environment. Wang et al. found a significant negative relationship between the school environment and aggression in students. It is expected that an inappropriate environmental conditions could have an association with the lack of students’ satisfaction, their poor connections with others, and poor school performance. Moreover, adolescents are more likely to express more deviant behaviors to compensate for such conditions.^66^

Self-efficacy is a key factor in learning and choosing strategies. Individuals with high self-efficacy can better overcome difficulties and obstacles. Hence, understanding self-efficacy can maintain health-promoting behaviors. ^67^ This study indicated that self-efficacy played a significant role in predicting aggressive behaviors. Consistent with the findings of this study, Lee et al. indicated that self-efficacy in individuals that played violent video games had a significant inverse relationship with aggressive behaviors.^68^ Also, the study by Sayarpoor et al. revealed a negative relationship between aggression and perceived self-efficacy in students.^10^ In this study, parents’ self-efficacy had no significant relationship with aggression in children. Despite the expression of parents for providing strategies to reduce aggression in children, such strategies lacked a proper ability to reduce aggression. in the study by Malm et al., parents’ self-efficacy had a significant negative relationship with aggression in children.^69^ In our study, self-efficacy was significantly and inversely associated with aggression in the students. Similarly, Veenstra showed that high levels of self-efficacy in teachers had a significant relationship with the low rate of bullying in students.^70^

In this study, a direct and significant relationship between perceived social norms about aggressive behaviors and aggression was reported. Accordingly, Jackson et al. ’s study on elementary school students indicated that aggression norms in the classroom social network played a significant role in aggressive behaviors. Therefore, in a class with aggression norm, the aggressive children are more accepted.^71^

Social support for children and adolescents consists of any informational, instrumental, evaluative and emotional support provided by teachers, family and friends.^72^ In our study, social support to control aggression did not show a significant relationship with student aggression, which could be due to the indirect effect of peer and adult support on students’ aggression as social support to control aggression can greatly affect aggressive behaviors by strengthening positive perceptions of the school environment, and scientific, social, and psychological development.^73^ In the study of Le et al., no significant relationship between social support of the family and bullying in students was found, that could be related to the inability of the family to help students solve the wide-ranging communicational problems and gaps between children and parents.^74^

This cross-sectional study lacks appropriate inference of the causal path of the independent and dependent variables. Also, the self-report method for aggressive behaviors, proposes the probability of error in the accurate estimation of this behavior. Future studies are needed to include measures based on more objective approaches such as the behavioral experience method.^75^ Factors influencing students’ aggressive behaviors based on a predeﬁned factors set by the SCT should be found. Future qualitative studies are suggested to identify silent factors influencing this phenomenon in the cultural and social context of Iran. 

## Conclusion

The Social Cognitive Theory is the appropriate framework for predicting aggression behaviors in children and adolescents. Observational learning of aggressive behaviors, perceived social norms about aggressive behaviors, self-efficacy to control own aggression, perceived situational, outcome expectations about the consequences of aggressive behaviors and outcome expectancies about the value which the student place on these consequences predicted 37.3% of changes in aggressive behaviors in the students. Aggression in children and adolescents can be prevented and reduced through designing and implementing educational interventions based on these factors.

**Acknowledgment**

The authors thank the students, parents, teachers, school staff and the Education Department of Kermanshah city for their participation and cooperation in this study.
